# Supplementary figures and images for: Mitochondrial introgression by ancient admixture between two distant lacustrine fishes in Sulawesi Island
Source: PLoS One. 2021 Jun 10;16(6):e0245316. doi: 10.1371/journal.pone.0245316 (PMC8192020; doi:10.1371/journal.pone.0245316)

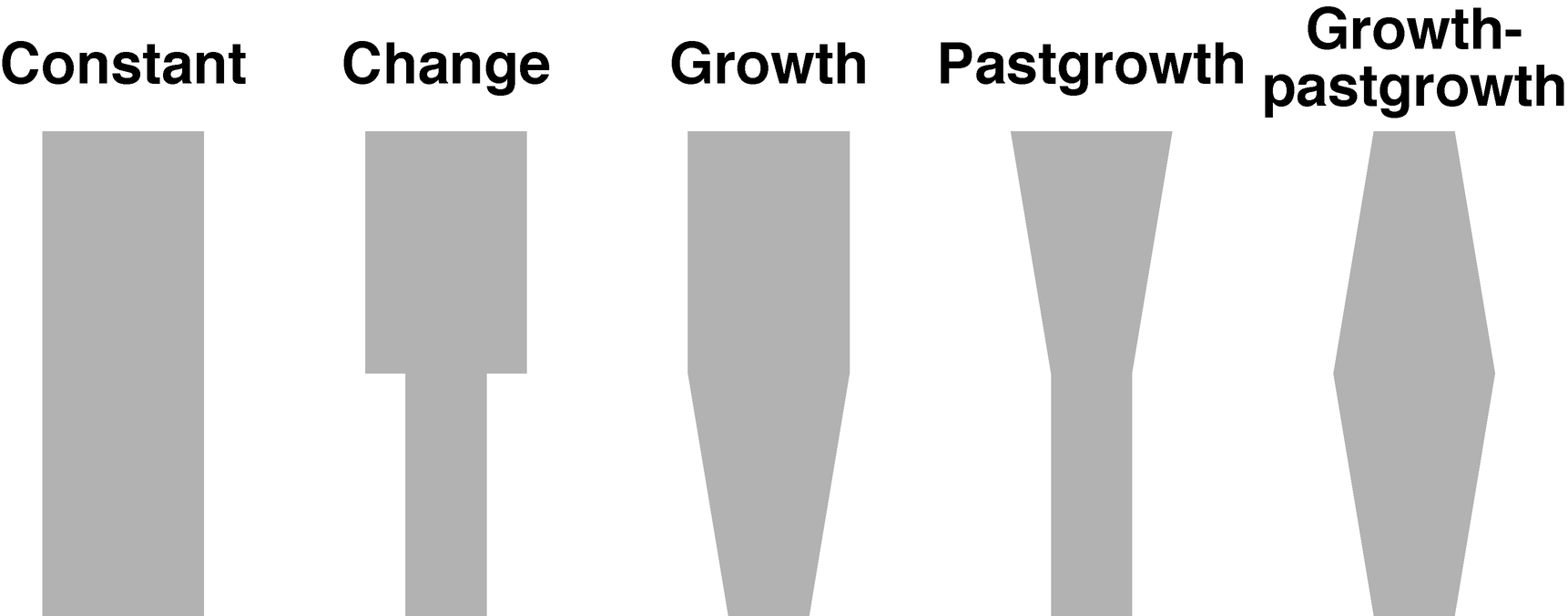

Supplement: S1 Fig — Note that growth was modeled to be exponential and not linear as depicted here. (TIF) [file pone.0245316.s001.tif]

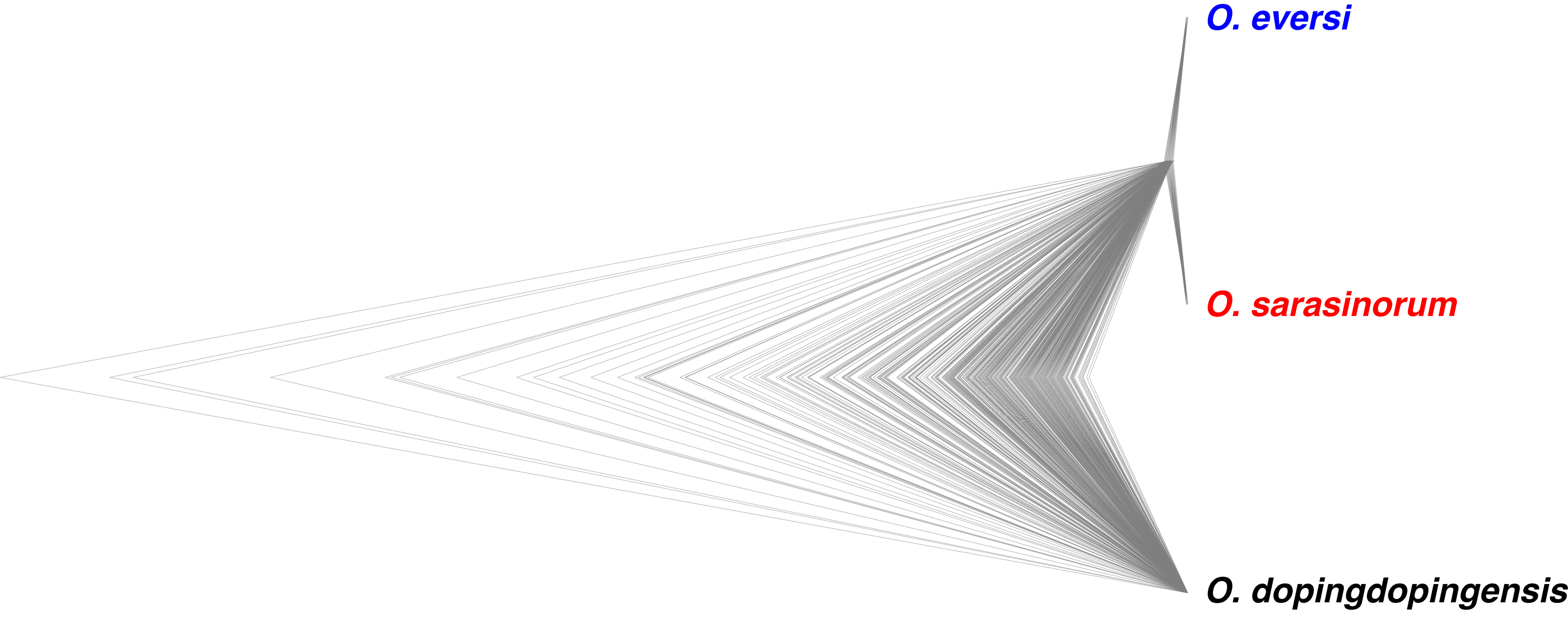

Supplement: S2 Fig — Thin lines represent individual species trees. (TIF) [file pone.0245316.s002.tif]

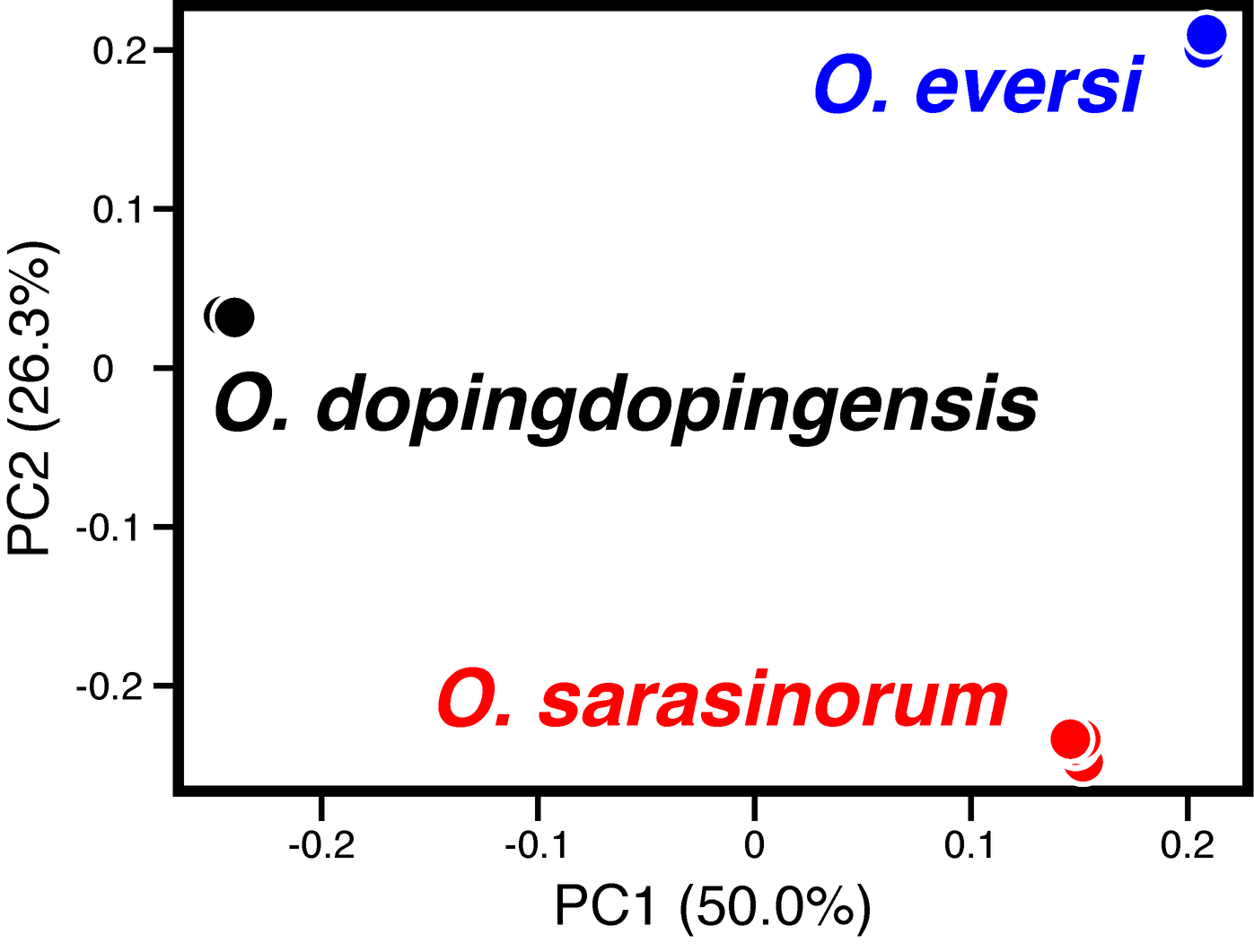

Supplement: S3 Fig — (TIF) [file pone.0245316.s003.tif]
