# Supplementary material for: Mitochondrial introgression by ancient admixture between two distant lacustrine fishes in Sulawesi Island
Source: PLoS One. 2021 Jun 10;16(6):e0245316. doi: 10.1371/journal.pone.0245316 (PMC8192020; doi:10.1371/journal.pone.0245316)
Supplement: S1 Table — (DOCX) [file pone.0245316.s004.docx]

**S1 Table. Sequencing reads deposited in the DDBJ Sequence Read Archive (accession number DRA011122).**

| Species | Individual ID | Run file | Number of raw reads |
| --- | --- | --- | --- |
| *Oryzias sarasinorum* | O_sar-01 | O_sar-01.fastq | 2,562,105 |
| *Oryzias sarasinorum* | O_sar-02 | O_sar-02.fastq | 3,254,079 |
| *Oryzias sarasinorum* | O_sar-03 | O_sar-03.fastq | 2,759,328 |
| *Oryzias sarasinorum* | O_sar-04 | O_sar-04.fastq | 1,661,594 |
| *Oryzias sarasinorum* | O_sar-05 | O_sar-05.fastq | 2,679,043 |
| *Oryzias sarasinorum* | O_sar-06 | O_sar-06.fastq | 2,866,601 |
| *Oryzias sarasinorum* | O_sar-07 | O_sar-07.fastq | 4,388,799 |
| *Oryzias sarasinorum* | O_sar-08 | O_sar-08.fastq | 3,840,872 |
| *Oryzias sarasinorum* | O_sar-09 | O_sar-09.fastq | 3,395,527 |
| *Oryzias sarasinorum* | O_sar-10 | O_sar-10.fastq | 3,346,696 |
| *Oryzias eversi* | O_eve-01 | O_eve-01.fastq | 1,640,068 |
| *Oryzias eversi* | O_eve-02 | O_eve-02.fastq | 4,541,875 |
| *Oryzias eversi* | O_eve-03 | O_eve-03.fastq | 3,385,891 |
| *Oryzias eversi* | O_eve-04 | O_eve-04.fastq | 1,702,133 |
| *Oryzias eversi* | O_eve-05 | O_eve-05.fastq | 1,855,401 |
| *Oryzias eversi* | O_eve-06 | O_eve-06.fastq | 1,708,631 |
| *Oryzias eversi* | O_eve-07 | O_eve-07.fastq | 4,248,663 |
| *Oryzias eversi* | O_eve-08 | O_eve-08.fastq | 1,859,212 |
| *Oryzias eversi* | O_eve-09 | O_eve-09.fastq | 1,439,810 |
| *Oryzias eversi* | O_eve-10 | O_eve-10.fastq | 2,763,098 |
