# Supplementary material for: Mitochondrial introgression by ancient admixture between two distant lacustrine fishes in Sulawesi Island
Source: PLoS One. 2021 Jun 10;16(6):e0245316. doi: 10.1371/journal.pone.0245316 (PMC8192020; doi:10.1371/journal.pone.0245316)
Supplement: S2 Table — S: number of segregating sites, H: number of haplotypes, Hd: haplotype diversity, π: nucleotide diversity, K: average number of nucleotide difference, Ho: per site observed heterozygosity averaged over samples, and Tajima’s D. Each value represents the average among 4,703 loci. Calculations were performed by DnaSP 6.X.X. (DOCX) [file pone.0245316.s005.docx]

**S2 Table. Genetic diversity of RAD locus.** *S*: number of segregating sites, *H*: number of haplotypes, *H*_d_: haplotype diversity, *π*: nucleotide diversity, *K*: average number of nucleotide difference, *H*_o_: per site observed heterozygosity averaged over samples, and Tajima’s *D*. Each value represents the average among 4,703 loci. Calculations were performed by DnaSP 6.12.03 [1]. Genetic diversity was generally lower in *Oryzias eversi* than in *O. sarasinorum*.

| Index | *Oryzias sarasinorum* | *Oryzias eversi* |
| --- | --- | --- |
| *S* | 0.22539 | 0.05422 |
| *H* | 1.21178 | 1.04890 |
| *H*_d_ | 0.05917 | 0.01744 |
| *π* | 0.00127 | 0.00039 |
| *K* | 0.06478 | 0.01966 |
| *H*_o_ | 0.00125 | 0.00040 |
| Tajima’s *D* | 0.03514 | 0.53134 |

**Reference**

**1** Rozas J, Ferrer-Mata A, Sánchez-DelBarrio JC, Guirao-Rico S, Librado P, Ramos-Onsins SE, Sánchez-Gracia A. DnaSP v6: DNA sequence polymorphism analysis of large datasets. Mol Biol Evol 2017; 34: 3299–3302. https://doi.org/10.1093/molbev/msx248 PMID: 29029172
