# Supplementary material for: Mitochondrial introgression by ancient admixture between two distant lacustrine fishes in Sulawesi Island
Source: PLoS One. 2021 Jun 10;16(6):e0245316. doi: 10.1371/journal.pone.0245316 (PMC8192020; doi:10.1371/journal.pone.0245316)
Supplement: S3 Table — (DOCX) [file pone.0245316.s006.docx]

**S3 Table. Support for one-population models defined in S1 Fig.**

(A) *Oryzias sarasinorum*

| Model | Number of parameters | log10-likelihood | Relative likelihood | ln-likelihood | AIC | ∆-AIC |
| --- | --- | --- | --- | --- | --- | --- |
| Pastgrowth | 4 | –5,851.739 | — | –13,474.127 | 26,960.878 | — |
| Change | 4 | –5,853.088 | 4.477×10^–2^ | –13,477.233 | 26,962.466 | 6.212 |
| Growth-pastgrowth | 5 | –5,855.293 | 2.793×10^–4^ | –13,482.310 | 26,974.621 | 18.367 |
| Growth | 4 | –5,856.097 | 4.385×10^–5^ | –13,484.162 | 26,976.323 | 20.069 |
| Constant | 2 | –5,881.505 | 1.714×10^–30^ | –13,542.666 | 27,089.331 | 133.077 |

(B) *Oryzias eversi*

| Model | Number of parameters | log10-likelihood | Relative likelihood | ln-likelihood | AIC | ∆-AIC |
| --- | --- | --- | --- | --- | --- | --- |
| Pastgrowth | 4 | –2,665.965 | — | –6,138.611 | 12,285.223 | — |
| Growth-pastgrowth | 5 | –2,666.159 | 6.397×10^–1^ | –6,139.058 | 12,286.116 | 0.893 |
| Change | 4 | –2,667.689 | 1.888×10^–2^ | –6,142.581 | 12,293.162 | 7.939 |
| Growth | 4 | –2,668.776 | 1.545×10^–3^ | –6,145.084 | 12,300.168 | 14.945 |
| Constant | 2 | –2,690.676 | 1.945×10^–25^ | –6,195.510 | 12,395.021 | 109.798 |
