# Supplementary material for: Mitochondrial introgression by ancient admixture between two distant lacustrine fishes in Sulawesi Island
Source: PLoS One. 2021 Jun 10;16(6):e0245316. doi: 10.1371/journal.pone.0245316 (PMC8192020; doi:10.1371/journal.pone.0245316)
Supplement: S4 Table — (DOCX) [file pone.0245316.s007.docx]

**S4 Table. Explanation of each parameter used in the coalescent-based demographic inference.**

| Parameter | Explanation of parameter |
| --- | --- |
| NPOP1 | Population size of *O. sarasinorum* |
| NPOP2 | Population size of *O. eversi* |
| NPOP3 | Population size of the unknown species |
| NDIV11 | Population size of *O. sarasinorum* at TDIV1 |
| NDIV12 | Population size of *O. eversi* at TDIV1 |
| NDIV22 | Population size of *O. eversi* at TDIV2 |
| NDIV23 | Population size of the unknown species at TDIV2 |
| NANC1 | Ancestral population size |
| NANC2 | Population size of the common ancestor between *O. eversi* and the unknown species at TDIV2 |
| TCHG1 | Time of the end of past population growth in *O. sarasinorum* (generation) |
| TCHG2 | Time of the end of past population growth in *O. eversi* (generation) |
| TDIV1 | Divergence time of *O. sarasinorum* and *O. eversi* (generation) |
| TDIV2 | Divergence time of *O. eversi* and the unknown species (generation) |
| TAD | Time of admixture from *O. eversi* to *O. sarasinorum* (generation) |
| ADMIX | Ratio of migrants from *O. eversi* to *O. sarasinorum* at TAD |
| MIGR12 | Migration rate from *O. eversi* to *O. sarasinorum* |
| MIGR21 | Migration rate from *O. sarasinorum* to *O. eversi* |
