# Supplementary material for: Mitochondrial introgression by ancient admixture between two distant lacustrine fishes in Sulawesi Island
Source: PLoS One. 2021 Jun 10;16(6):e0245316. doi: 10.1371/journal.pone.0245316 (PMC8192020; doi:10.1371/journal.pone.0245316)
Supplement: S5 Table — (DOCX) [file pone.0245316.s008.docx]

**S5 Table. Intraspecific (diagonal) and interspecific (bottom left) average pairwise genetic distance (p-distance) based on the mitochondrial sequences.**

|  | *O. sarasinorum* | *O. eversi* |
| --- | --- | --- |
| *O. sarasinorum* | 0.0185 | — |
| *O. eversi* | 0.0201 | 0.0004 |
