# Supplementary material for: Mitochondrial introgression by ancient admixture between two distant lacustrine fishes in Sulawesi Island
Source: PLoS One. 2021 Jun 10;16(6):e0245316. doi: 10.1371/journal.pone.0245316 (PMC8192020; doi:10.1371/journal.pone.0245316)
Supplement: S6 Table — (DOCX) [file pone.0245316.s009.docx]

**S6 Table. Inferred maximum-likelihood parameters for each of ADM2_model and DGF_model.**

| Parameter | ADM2_model | DGF_model |
| --- | --- | --- |
| NPOP1 | 81,308 | 155,629 |
| NPOP2 | 4,899 | 13,341 |
| NPOP3 | 595,125 | — |
| NDIV11 | 179,473 | 33,764 |
| NDIV12 | 669,885 | 349,684 |
| NDIV22 | 110,235 | — |
| NDIV23 | 420,978 | — |
| NANC1 | 371,849 | 470,905 |
| NANC2 | 133,596 | — |
| TCHG1 | 47,077 | 7,179 |
| TCHG2 | 5,711 | 13,661 |
| TDIV1 | 109,151 | 60,373 |
| TDIV2 | 23,370 | — |
| TAD | 6,083 | — |
| ADMIX | 0.04260 | — |
| MIGR12 | — | 2.5145×10^–23^ |
| MIGR21 | — | 6.7608×10^–23^ |
