# Supplementary material for: Mitochondrial introgression by ancient admixture between two distant lacustrine fishes in Sulawesi Island
Source: PLoS One. 2021 Jun 10;16(6):e0245316. doi: 10.1371/journal.pone.0245316 (PMC8192020; doi:10.1371/journal.pone.0245316)
Supplement: Attachment — Submitted filename: Response_to_Reviewers.docx [file pone.0245316.s016.docx]

Response to Reviewers

Reviewer #1: Mitochondrial introgression by ancient admixture between two distant lacustrine fishes in Sulawasi Island

Mizuki Horoiwa et al.

The phylogenetic relationship between the different species of the Orysias genus was studied in a manuscript (2015) and it is clear that the mitochondrial DNA pattern between the two species Orysias sarasinorum (endemic Lake Lindu) and O. eversi (Tilanga fountain), which are approximately 190 km apart, was surprising (O. eversi mitochondrial haplotype in O. sarasinorum population). In this study, Horoiwa et al. focus on the evolutionary process responsible of the observed pattern. They test two evolutionary scenarios : incomplete lineage sorting versus introgressive hybridization between the two endemic species O. sarasinorum and O. eversi using the NADH dehydrogenase subunit 2 mitochondrial genes and 1552 SNP.

It is an interesting research based on species belonging to the genus of the well-known species model O. latipes. The final result is clear (hybridization in a “recent” past between the two species). The scientific question is clearly exposed and the results are based on a rigorous protocol and a good analysis.

Particular comments:

1) The figures in the pdf file are extremely difficult to read. I need to increase up to 200%.

2) It will be interesting to introduce the number of each line in the manuscript.

We collected them accordingly.

Specific comments:

Introduction

3) “Another possibility is incomplete lineage sorting”: could the authors indicate the mean genetic distance between/within group for the two species based on cytochrome b. The information could be a little bit redundant with the Figure 1, but in my opinion this second hypothesis make sense only if the genetic divergence is low (<0.05 on cyt b in teleosteans, as observed in the tree figure 1) otherwise the hypothesis 1 (hybridization) is sufficient.

We calculated intra- and interspecific pairwise genetic distance (p-distance) based on the mitochondrial sequences, as suggested (see L161–162, L285–286, and S5 Table).

Material and Methods

Field collections

4) Page 5. How collected specimens using beach seine in one place, one time was representative to the diversity of the different species? The 10 specimens could belong to the same “shoal”/family and genetically highly related? Could you give an idea of the population size for the different species?

The demographic inference by fastsimcoal2 revealed that the population size of *O. sarasinorum* and that of *O. eversi* were estimated about 40,700–86,300 and 2,500–7,200, respectively (see Fig 5 and S6 Table). These estimations are intuitive; the former inhabits in a large lake (34.5 km^2^) while the latter in a small fountain (~20 m wide). Because *Oryzias* is not social, we guess that the possibility that the collected individuals were relatives with each other are low. We mentioned this in the revised manuscript (L138–142).

ddRAD sequencing

5) Page 5. “the reads sequencing were deposited in DDBJ Sequence Read Archive”. It is a good practice, I appreciate.

We also appreciate this comment.

6) Page 5. Could you add the two restriction enzymes in the text: BglII and EcoRI.

Yes, they were *Bgl*II and *Eco*RI. We added the information of the restriction enzymes (L170).

7) Page 5. Could you indicate the quality score Q of the reads that you used, Q>30?

It was Q>20. We added more information about the filtering of raw reads (L178–181).

8) Page 5. Could you indicate the number of raw reads for each specimen?

We indicated the number of raw reads in S1 Table.

9) Page 5. I understand that the authors prefer to use a calibrated pipeline, however we have now the version 2.55 (https://catchenlab.life.illinois.edu/stacks/).

We appreciate this updated information. We had no problem using the calibrated pipeline.

10) Page 6. “Loci deviated from Hardy-Weinberg equilibrium… “It will be interesting to give some diversity indices such observed heterozygozity, private alleles. Considering that 10 specimens were collected, we need to have an idea about the polymorphism of the sampled specimen.

We calculated those indices for each species (S2 Table).

11) Page 6. Considering the 1,552 SNP sites could you indicated the % of missing data for each specimen, within species and between species.

No missing data was allowed in the present dataset. We clarified this point in the revised manuscript (L187–188).

12) How many SNP are diagnostic between the two species (as an example “A” fixed for O. sarasinorum and “G” fixed for O. eversi) ?

Among the 1,552 SNPs, 290 SNPs were diagnostic. We mentioned it in the revised manuscript (L206–207).

13) Could you indicate the number of transition and the number of transversion for the 1,552 SNP.

Among the 1,552 SNPs, 887 and 665 SNPs were transitional and transversional substitutions, respectively. We mentioned it in the revised manuscript (L195).

Phylogenetic analyses/population structure/Coalescence based demographic inference

14) This part is well written, with numerous information on the parameters used and tested hypotheses.

We appreciate this comment.

15) Page 7. “We used a synonymous substitution rate of 3.5 10 x 10-9 per site per generation” Why synonymous ? The mapping of the SNP was done on coding sequence only ?

It was our mistake. It was not “a synonymous substitution rate” but “a mutation rate”. We collected it accordingly (L248 and L267). This is the de novo mutation rate in a cichlid parent–offspring trio obtained from whole-genome sequencing data (Malinsky et al. 2018).

16) Is it possible to use DIYABC (ref 18) based on various prior distributions to estimate the posterior distribution of the substitution rate (median of the posterior distribution)?

We appreciate this suggestion. However, we would like to estimate the mutation rate of this group not by model approaches but by empirical observations. Indeed, we are planning to conduct a laboratory experiment to estimate the mutation rate of this group using parent–offspring trios. We hope that more plausible time estimations will be possible soon, when that mutation rate becomes available.

17) Depending to the % of missing data, it will be important to test the impact on the population structure and Coalescence based demographic inference (i.e. if the % is higher than 30%).

In my opinion if the % of missing data is lower than 30% it is not necessary.

As above, no missing data was allowed.

Discussion

18) Page 11. Assuming the generation time… was to high. I agree with the authors, however it is important to have a better idea of the substitution rate parameter (and to have an idea of the impact of missing data, if missing data there are).

As above, we are planning to conduct a laboratory experiment to estimate the mutation rate of this group using parent–offspring trios. We hope that more plausible time estimations will be possible soon, when that mutation rate becomes available.

19) Another question is how genetically highly related specimen for each species could impact the analyses and the conclusion?

As we mentioned above, the population size of *O. sarasinorum* and that of *O. eversi* were estimated about 40,700–86,300 and 2,500–7,200, respectively (see Fig 5 and S6 Table). We guess that the possibility that the collected individuals were relatives with each other are low, because *Oryzias* is not social.

Reviewer #2: This is an interesting case. The team of authors focuses on a previously known case of paraphyly in mitochondrial haplotypes of Oryzias sarasinorum, ricefish endemic to Lake Lindu in Sulawesi. They extend the mitochondrial data, show that the two species analyzed (O. sarasinorum, O. eversi) are respectively monophyletic in their nuclear genomes compared to O. dopingdopingensis, and suggest possible scenarios that might explain the case.

While I consider analyzing the case of paraphyly very interesting, the manuscript suffers from substantial discrepancies among actual findings and conclusions, and also from limited coverage of the relevant literature.

Major issues:

1) The scenario of “three major tectonic subdivisions” (Introduction; cited are Hall 2009, 2011, Sparkman & Hall 2010) does not reflect the current state of knowledge: Substantial revisions of that concept have been proposed by Hall & Nugraha 2017, and Nugraha & Hall (2018). The authors cite one of these more recent references latter in the Discussion (as Nagrahaa & Hall 2018), however without incorporating or discussing the core framework of paleo-islands and expansions. As the geographic scenario proposed here rests on an apparently outdated geological background, the evolutionary implications proposed here also appear not valid.

We appreciate this essential suggestion. We cited Nugraha and Hall (2018) (we could not find Hall and Nugraha 2017) in the Introduction section and mentioned that large portions of land have been uplifted since Pliocene (over the last 2–3 Myr) based on their findings (L62).

2) Likewise, the assumption of a “lake or lake system” scenario (Discussion), and its possible fragmentation, is largely speculative, based not on geological or limnological data, but exclusively on a genomic study on ricefish speciation in Lake Poso, published by the same group of authors (Sutra et al. 2019). Core presumptions of the discussion are not covered by published studies or data, this is not acceptable.

We discussed in the Discussion section that the “lake or lake system” scenario is quite likely, because the Plio-Pleistocene uplift of large portions of land, which was demonstrated by Nugraha and Hall (2018), may have simultaneously changed river and lake systems on this island drastically (L369–371). We also think that the fossil species (†*Lithopoecilus brouweride*) is a strong support to this scenario from geology.

3) Tilanga Fountain (the site where Oryzias eversi is endemic) is not a lake (Abstract, Discussion), it’s a 4 m deep and 10 m wide karst pond, an extension of a stream (see the original description of O. eversi). The present manuscript implies hybridization among lake endemics, which is not the case. Oryzias eversi should accordingly also not be called a “lacustrine species” (first header of the Discussion).

We went to Tilanga Fountain twice, and we found that this fountain is not an extension of a stream, that is, we found no surface connection with a stream, and that there is no water current like in a river. In other words, this fountain is completely isolated at least from any surface water systems, although we found water gushing out from underground, indicating an underground water system. That’s why we call *O. eversi* a “lacustrine species”, as an antonym of a “riverine species”, in this study. We clearly mentioned this in the revised manuscript (L138–142).

4) The selection of references is strongly biased towards the own work of the team of authors. It ignores previous findings on mitochondrial introgression and hybridization in Sulawesi lake fishes (e.g., Herder et al. 2006 Proc Roy Soc B, Schwarzer et al. Hydrobiologia 2008), as well as other work on Sulawesi ricefishes (to be found in the eLIFE review by Schwarzer & Hilgers 2019). A sound interpretation should take the relevant spectrum of reference into consideration.

We cited those non-adrianichthyid studies as other examples of mitochondrial introgression in the Discussion section (L396–407). We also included Hilgers and Schwarzer (2019) in the Introduction section as a study focusing on the adrianichthyid diversity on Sulawesi (L66–67).

In sum, the main line of discussion appears not valid when considering the points raised above. What remains is an interesting case of past genetic exchange among two populations of fishes nowadays separated by a distance of ca. 190 km, that is in need for plausible explanation.
