# Supplementary material for: Mitochondrial introgression by ancient admixture between two distant lacustrine fishes in Sulawesi Island
Source: PLoS One. 2021 Jun 10;16(6):e0245316. doi: 10.1371/journal.pone.0245316 (PMC8192020; doi:10.1371/journal.pone.0245316)
Supplement: Attachment — Submitted filename: Response_to_Reviewers.docx [file pone.0245316.s017.docx]

Response to reviewer’s comments

1) There is to the best of my knowledge no geological evidence that suggests the existence of a “big lake or lake system until the Pliocene” (l. 311-312), the same applied to the hypothesis of “several lakes or lake systems” (l. 312-313). The authors cite a phylogenetic paper on ricefishes from their own group for justifying this statement, but still fail in providing independent evidence. This is relevant, as the interpretation of the results largely collapses, once this speculation is removed.

We showed a photo of †*Lithopoecilus brouweri* to a paleontologist (Dr. Toshihiro Yamada, Osaka City University) to consult him about the environment where it fossilized. According to him, this fossil specimen was contained in well-laminated mudstone which should have deposited in lacustrine environments, and it is consistent with the palaeogeographical reconstruction of this island. We think that this is strong evidence for our view that there was a lake or lake system there until Pliocene. We added this information in the revised manuscript (L339–342).

2) The assumption that the fossil “…†Lithopoecilus brouweri is the common ancestor of O. sarasinorum and O. eversi…” (l. 335-336) is likewise pure speculation. The morphology of this fossil fish remains largely unclear: Frickhinger 1991 (cited here) illustrates the fossil, but gives little more information than that this is a small and slender fish with large eyes and a pointed head; Parenti 2008 (the second source cited here) says that she did not see the specimen, but that she sees no reason to contradict Beaufort 1934, who said that it is intermediate between Oryzias and Adrianichthys. In sum, the knowledge of †Lithopoecilus brouweri morphology is very limited. It can be said that a ricefish that was considered in 1934 being similar to other Sulawesi ricefishes occurred in the area in the Miocene – that’s it. Anything else would require new studies of this fossil specimen.

Parenti (2008) stated that “I have not examined the fossil and cannot place it unambiguously in either *Oryzias* or *Adrianichthys* (p.592)”, implying that she accepted the Beaufort’s observation that †*Lithopoecilus* is intermediate between the two genera. Either way, we weakened our argument by stating “we think that †*L. brouweride* is the common ancestor of *O. sarasinorum* and *O. eversi*, but further examinations of this fossil species are necessary” (L337–339).

Further, I do not share the view that tiny Tilanga fountain is an environment that is to be termed “lacustrine”. Lacustrine refers to a “lake environment”, whereas the Tilanga fountain is a tiny pool, connected to groundwater, with a small overflow. The point I raise is however less that of the terminology, it questions if the habitat is from its properties somehow comparable to “real” lakes, such as Lake Lindu: Substantial and long-lived, largely stagnant waters. To my understanding, there is also no evidence that the Tilanga fountain can be seen as a leftover of a lake – its simply a fountain, and we do not know more about that habitat so far.

Because our point in this study is that a large ancient lake(s) in central Sulawesi was fragmented into several small ones, we would like to keep the term “lacustrine” in this manuscript. So, we decided to compromise with the referee’s point as follows. First, we deleted the definition of “lacustrine” from the materials and methods section (L122) and used “lacustrine and/or pond” when the mention there was limited only to *O. sarasinorum* and *O. eversi* (L50 and L299). In contrast, we used “lacustrine” where our mention was about adrianichthyids or other lacustrine organisms in central Sulawesi in general (L52, L73, L97, L98, L312, L321, and L368). We would like to keep “lacustrine” in the title, because this title includes our message as above.

Having raised these points of criticism, I would like to express that I would in fact like seeing these interesting genetic results published, but with an interpretation that is based on the existing body of evidence. I feel that the idea of interconnection of the two sites, Tilanga and Lindu, by a lake or several lakes, could be proposed as one possible scenario – stating clearly that there is so far no further evidence available. And I would expect a critical discussion, including alternative scenarios. If this is done, the present paper will also remain valid if it should turn out later, that that there was in fact no lake connection among Tilanga and Lindu.

In this study, we compared two alternative scenarios, i.e., incomplete lineage sorting (ILS) and ancient admixture, using model-based genetic analyses. The results clearly supported the ancient admixture scenario rather than ILS. Although we agree that geological studies would be needed to demonstrate our hypothesis, the existence of †*Lithopoecilus brouweri* seems to be strong enough to support our hypothesis for the moment. In the present circumstance, there is no alternative scenario or hypothesis available to explain our results, and even if any, there would be no foundation.

The same applies to the Tilanga habitat: Terming this “lacustrine” raises the expectation that these are two populations of lake fish, which is not the case. Saying openly that this is a “fountain” would appear to me even more interesting, and clearly correct.

As above, we used “lacustrine and/or pond” when our mention was limited to *O. sarasinorum* and *O. eversi* (L50 and L299).
